# COVID‐19‐related fears and information frequency predict sleep behavior in bipolar disorder

**DOI:** 10.1002/brb3.2182

**Published:** 2021-08-19

**Authors:** Frederike T. Fellendorf, Eva Z. Reininghaus, Michaela Ratzenhofer, Melanie Lenger, Alexander Maget, Martina Platzer, Susanne A. Bengesser, Armin Birner, Robert Queissner, Carlo Hamm, Rene Pilz, Nina Dalkner

**Affiliations:** ^1^ Department of Psychiatry and Psychotherapeutic Medicine Medical University of Graz Graz Austria

**Keywords:** bipolar disorder, COVID‐19 pandemic, Pittsburgh Sleep Quality Index, sleep

## Abstract

**Introduction:**

The coronavirus disease (COVID‐19) pandemic and consequent restrictions including social distancing had a great impact on everyday life. To date, little is known about how the restrictions affected sleep, which is commonly disturbed in bipolar disorder (BD). The aim of this study was to elucidate sleep patterns during the pandemic in Austrian BD individuals.

**Methods:**

An online survey assessed sleep with the Pittsburgh Sleep Quality Index (PSQI) and COVID‐19‐associated attitudes, fears, and emotional distress of 20 BD individuals and 19 controls (HC) during the pandemic. The survey was conducted in April 2020, when very strict regulations were declared, and repeated in May, when they were loosened.

**Results:**

Individuals with BD reported overall poor sleep according to PSQI sum at both time points. Subjective sleep quality, sleep latency, daytime sleepiness, and PSQI sum were worse in individuals with BD than in HC. Individuals with BD informed themselves more frequently about pandemic‐related topics. Higher information frequency and more COVID‐19 fears (about the virus, own infection, contracting others) correlated with worse PSQI values. Regression models found in BD group that higher information frequency as well as higher COVID‐19 fears in April predicted worse sleep characteristics in May, in particular subjective sleep quality, sleep duration, sleep efficiency, and daytime sleepiness.

**Conclusion:**

As sufficient sleep duration and quality are essential for well‐being and particularly important for vulnerable BD individuals, it is important that information about the pandemic is gathered to a reasonable extent and mental health professionals include COVID‐19‐related fears when currently treating BD.

## Graphical abstract

The online survey showed that individuals with bipolar disorder (BD) reported overall poor sleep in comparison with healthy controls during total lockdown due to the COVID‐19 pandemic as well as when restrictions were loosened. Individuals with BD informed themselves more frequently about pandemic‐related topics and higher information frequency and more COVID‐19 fears (about the virus, own infection, contracting others) correlated with worse sleep. Regression models found in BD group that higher information frequency as well as higher COVID‐19 fears in April predicted worse sleep characteristics in May, in particular subjective sleep quality, sleep duration, sleep efficiency, and daytime sleepiness.

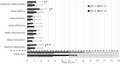

## 1 INTRODUCTION

An unbalanced sleep–wake rhythm can complicate the course of illness episodes of bipolar disorder (BD) and can trigger relapse. Sixty‐nine percent of individuals with BD suffer from sleep disturbances (Laskemoen et al., 2019). Beside manic and depressive episodes, sleep disturbances can also occur in euthymia and are associated with lower global and cognitive functioning (Laskemoen et al., 2019, 2020). Clinical symptoms of BD, such as sleep disorders, are affected by circadian rhythms, but disturbed sleep–wake rhythms can also influence or trigger illness episodes (Lewis et al., 2017). In turn, both depressive and manic episodes can lead to sleep disturbances in the early stages. Different levels of vulnerability for BD are assumed to exist due to biological processes such as genetics, unstable biological rhythms (Bengesser et al., 2018), inflammatory processes (Goldstein et al., 2009), imbalance of central neurotransmitters, and psychosocial influences such as life events, personality, and family environment. Additional chronic or current psychosocial stressors such as trauma, separations, job loss, and abuse of psychotropic substances, but also unstable sleep–wake rhythms, may provoke an exacerbation of (symptoms of) the disorder. The severe acute respiratory syndrome coronavirus‐2 (SARS‐CoV‐2) pandemic, which causes the novel coronavirus disease (COVID‐19), and its consequences including social distancing and self‐isolation, might be severe triggers for the development of BD illness episodes. There are preliminary studies that show elevated psychosocial distress and lifestyle changes during the pandemic in BD subgroups (Van Rheenen et al., 2020).

To contain the spread of the pandemic, governments across the world have passed new laws and restrictions. Like many other countries, Austria imposed a lockdown, enforced social distancing as well as home office wherever possible. From March 15th 2020 until the end of April, the lockdown included all nonessential shops, restaurants, universities, schools, nurseries, and gyms. Additionally, public events or meetings with people from different households were not allowed. Everybody except key workers had to work from home. It was compulsory to wear face masks in supermarkets and to keep a distance of at least one meter. In case of transgressions, fines had to be paid. On the first of May, the restrictions were loosened. Gatherings of up to ten people were allowed, and nonessential shops, hairdressers, and leisure venues reopened. By the end of May, restaurants, schools, and nurseries opened again and events with up to 100 people were allowed.

Very recent studies, highlighting the effects of the pandemic in 2020, found that the pandemic itself, but also the statutory restrictions, has been shown to be associated with multiple psychosocial symptoms. This extraordinary situation correlates with feelings of uncertainty, general low well‐being, stress, depression, somatization, anxiety, sleep disturbances, changes in alcohol and nicotine intake, and fear of infection (Pérez‐Fuentes et al., 2020; Stanton et al., 2020). Social distancing and quarantine can result in confusion, anger, frustration, and boredom, being misinformed as well as financial problems (Brooks et al., 2020). These factors may lead to behavioral and lifestyle changes, in lifestyle, for example, the disruption of the sleep–wake rhythm. However, the manifestation of symptoms depends on previous mental (Vindegaard & Benros, 2020) and somatic diseases, personality structure, resilience, and individual coping mechanisms (Pfefferbaum & North, 2020). Due to these reasons, the COVID‐19 pandemic directly and indirectly leads to exceptional challenges for mental health services (Fatke et al., 2020).

To date, very little is known about sleep during such an extraordinary situation as the COVID‐19 pandemic. It is discussed that uncertainty, worries about one's own and others' health and the situation, changes in daily routines, omission of sports and hobbies, working from home, home‐schooling, financial problems, and other pandemic‐related stressors have a negative impact on sleep (Altena et al., 2020; Morin et al., 2020). Sleep is essential for the human organism. Sufficiently long and good sleep is necessary for recovery, cell growth, synaptogenesis, and cognitive function (Frank & Heller, 2018). An individual's required sleep time is commonly between 7 and 9 hr. However, duration or quality of sleep is often and easily disturbed. Insomnia with problems falling asleep, staying asleep, and early morning awakening and hypersomnia are possible disorders that occur in isolation or in the context of various mental illnesses (Saletu‐Zyhlarz et al., 2013). In general, sleep is influenced by daylight, light from electronic devices, particularly in the evening, melatonin metabolism, daily routines, physical activity, alcohol and diet as well as stress. In addition, poor sleep quality was associated with increasing age, female sex, migration background, unemployment, physical inactivity, somatic health problems, mental illness, and again stress (Azevedo Barros et al., 2019). Social isolation and loneliness are related to poor sleep too, presumably mediated by depression and stress (Wakefield et al., 2020). There are contradicting findings about the impact of social interactions on sleep quality. Some research indicated that social interactions and social support are related to better sleep quality (Kent de Grey et al., 2018); however, a Chinese study group found that fewer social contacts during the pandemic improved sleep quality by reducing anxiety and stress (Xiao et al., 2020). Moreover, time spent occupying oneself with pandemic‐related topics might be relevant for feelings, lifestyle, and sleep. On the other hand, good sleep can have a preventive effect against psychosocial consequences. It was shown that people living in Wuhan, who had better sleep quality and less early awakenings in times of the severe pandemic, had an decreased risk of developing post‐traumatic stress symptoms (Liu et al., 2020). Assessing sleep disturbances as critical pathophysiological elements in BD could help to gain a deeper understanding of the psychological consequences on the course of BD due to the COVID‐19 crisis.

To date, no trial has investigated sleep quality and sleep patterns during the COVID‐19 pandemic in the especially vulnerable group of individuals with BD. Therefore, the present study aimed to determine whether (a) sleep was different in individuals with BD compared to mentally healthy people during COVID‐19 restrictions, (b) sleep patterns changed in individuals with BD when transitioning from strict distancing regulations to loosened regulations, (c) sleep is related to how frequently individuals access information about COVID‐19‐related topics, and (d) sleep is associated with COVID‐19‐related fears and attitudes.

## 2 METHODS

### 2.1 Procedure and participants

The survey was conducted at the Medical University of Graz, Department of Psychiatry and Psychotherapeutic Medicine as a part of the ongoing multicenter BIPLONG study. The BIPLONG study aims to explore the relationship between BD and obesity, metabolism, lifestyle, and cognitive function in a longitudinal setting. Individuals with BD—diagnosed with the Structured Clinical Interview for DSM‐IV (SCID‐I)—as well as individuals without a mental disease (healthy controls, HC) are invited to participate in the study for up to thirteen visits every 6 months in the dedicated outpatient center for BD of the Medical University Graz. Individuals with BD were either former inpatients or outpatients of the dedicated outpatient center for BD. HC were recruited from the general population via written invitations and word of mouth (circle of acquaintances, medical students, clinical personnel staff). HC were screened for psychiatric diseases with a short screening questionnaire based on the SCID. From 9 April to 28 April 2020 (t1), at the time of the complete lockdown, an additional online survey was sent out to study participants using the software LimeSurvey (www.limesurvey.org; Limesurvey GmbH). The same survey was rerun from 5 May to 4 June 2020 (t2), when the restrictions were loosened. The study has been approved by the local ethics committee (Medical University of Graz, Austria; EK‐number: 25–335 ex 12/13) in compliance with the current revision of the Declaration of Helsinki, ICH guideline for Good Clinical Practice and current regulations.

The current investigation included 20 individuals with BD (10 females, 10 males) as well as 19 HC (14 females, 5 males) who completed the survey at both time points. Individuals with BD were outpatients of the special unit for BD, and like HC, had to be of legal age and had given written informed consent prior to their participation in this study. The participation of the online survey was voluntary and pseudo‐anonymously used the participant code of the former study participation.

### 2.2 Materials

The *Pittsburgh Sleep Quality* *Index* (PSQI) by Bysse et al. (1989) is a self‐rated questionnaire, which assesses sleep quality and disturbances over a 1‐month time interval. The questionnaire consists of 19 items, which generate seven components: subjective sleep quality, sleep latency, sleep duration, habitual sleep efficiency, sleep disturbances, use of sleep medication, and daytime sleepiness. Each component scores from 0 (“no difficulty”) to 3 (“severe difficulty”). A total PSQI score (range 0–21) of more than 5 reaches a diagnostic sensitivity of 89.6% and specificity of 86.5% (*kappa* = 0.75; *p* ≤ .001) in distinguishing good and poor sleepers (Bysse et al., 1989), whereas higher scores indicate worse sleep quality. Despite the prevalence of sleep complaints among psychiatric patients, few questionnaires have been specifically designed to measure sleep quality in clinical populations. The PSQI was evaluated over an 18‐month period in a mentally healthy cohort, as well as in individuals with depression and in individuals with sleep disorders. Acceptable measures of internal homogeneity, consistency (test–retest reliability), and validity were obtained.

The *Beck Depression Inventory‐II* (BDI‐II), German version by Kühner et al. (2007), is a self‐rated questionnaire with 21 items, which assesses depressive symptomatology. The total score ranges from 0 to 63 points, whereas a score of 18 points or more indicates clinical depression. There is an internal consistency with Cronbach's *α* ≥ 0.84 and a reliability of *r* ≥ 0.75.

The *Altman Self‐Rating Mania* *Scale* (ASRM) by Altman et al. (1997) is a 5‐item scale for assessing mood, self‐confidence, sleep disturbances, speech, and activity level over a 1‐week period. Each question can be rated with 0 to 4 points, and a total score above 5 correlates with manic symptoms.

The self‐constructed COVID‐19 questionnaire in German collected data about sociodemographic variables, living situation, activities, hobbies, and use of medication. Moreover, it inquired about COVID‐19‐related attitudes, lifestyle, and fears as well as trust in regulations and emotional distress due to physical and social distancing over the last week. Inter alia, to assess *information frequency* participants were asked to rate how often they inform themselves about the pandemic and associated topics on a 6‐point scale (1 = “less than once a week,” 2 = “1–3 times per week,” 3 = “3–6 times per week,” 4 = “1–3 times per day,” 5 = “4–6 times per day,” and 6 = “more than 6 times a day”). *COVID‐19 fears* were assessed by a mean index of “*How strongly do you rate your concerns and fears about the coronavirus?”, “How strongly do you rate your fear of contracting the coronavirus?” and “How strongly do you rate your fear of infecting others with the coronavirus?”*. Each question could be answered with 0 (“no fear”) to 10 (“very strong fear”). The sum was calculated and divided by three. *Emotional distress* was surveyed by five items with a 6‐point rating scale from 0 (“not at all”) to 5 (“very strong”). The items included the questions “*Social distancing makes me feel lonely/bored/frustrated/hopeless/anxious.”* The index was calculated by the sum of the questions divided by five.

### 2.3 Statistical analyses

All analyses were performed with the IBM Statistical Package for Social Sciences (SPSS), version 25.0. Chi‐square test (nominal data), *t* tests (metric data), and Mann–Whitney U tests (ordinal data) were conducted to test for differences between the BD and HC group in descriptive variables. Multivariate covariance analyses (MANCOVAs) were used to calculate the differences between the BD and HC group in the PSQI components at t1 and t2, whereas age and sex were used as covariates due to their known clinical impact and statistical group differences. Paired‐sample *t* tests or Wilcoxon tests were used to analyze differences between the two time points. Partial correlations with age and sex as covariates were performed to identify associations of the PSQI components with COVID‐19 fears and emotional distress variables. As the information frequency is an ordinal variable, Spearman correlations were used for this item. Correlation analyses were calculated separately for the BD and HC group. The items of t1 correlating significantly with items concerning sleep of t2 as well as age and sex were used in regression models to analyze the variance of prediction of the sleep components. Error probabilities below 0.05 were accepted due to the clinical nature of the study. Bonferroni corrections were adjusted in correlation and regression analyses for the seven PSQI components (0.05/7 tests = 0.0071).

## 3 RESULTS

Table 1 displays the sociodemographic characteristics and differences in current mood as well as COVID‐19‐associated fears and attitudes in April and May 2020. Individuals with BD were significantly older, had a higher body mass index, and were less often in an employment relationship compared to HC. No participant was tested positive for SARS‐CoV‐2, lived together with a person tested positive or was in quarantine until survey participation. Individuals with BD scored significantly higher in the BDI‐II, informed themselves more frequently and had more COVID‐19‐related emotional distress than HC at t1. At t2, there were significant differences between the groups in BDI‐II and ASRM. The means of BDI‐II, information frequency, COVID‐19 fears, and emotional distress decreased in both groups from April to May, whereas the ASRM score increased in the BD group. Importantly, only the information frequency change was statistically significant (BD group: *Z* = 3.226, *p* = .001; HC group: *Z* = −2.585, *p* = .010). All values of BDI‐II and ASRM were in the nonpathological range (see Table 1).

**TABLE 1:** Sociodemographic characteristics and differences in mood symptomatology as well as COVID‐19‐related attitudes and fears of individuals with BD and HC

	BD (*n* = 20)	HC (*n* = 19)	Statistics	*p* value
Sex	50% male	26.3% male	*x*^2^ = 2.309	.129
Age in years (M, *SD*)	49.35 (±15.55)	33.05 (±9.66)	*t*_37_ = 3.906	**<.000** **
Diagnosis	60.0% BD I 40.0% BD II	–		
t1				
Professional activity			*x^2^* = 19.988	.**003** **
Working as before	0%	26.3%		
Working in home office	25.0%	52.6%		
Invalidity pension	50.0%	0%		
On demand/reduced hours	10.0%	5.3%		
Jobless due to pandemic	5.0%	0%		
Jobless as before pandemic	5.0%	0%		
In education	5.0%	15.8%		
BDI‐II	15.45 (±10.74)	2.95 (±2.50)	*t*_21.164_ = 5.062	**<.000** **
ASRM	0.95 (±2.28)	0.53 (±1.22)	*t*_37_ = 0.718	.472
Information frequency^†^	4.55 (±0.89) MR: 23.55	3.79 (±0.86) MR: 16.26	*U* = 119.000	.**047** *
COVID‐19 fears^‡^	4.45 (±2.69)	3.58 (±2.00)	*t*_37_ = 1.121	.269
Emotional distress^§^	1.56 (±1.16)	0.76 (±0.51)	*t*_26.372_ = 2.819	.**009** **
t2				
BDI‐II	12.20 (±11.69)	2.21 (±2.10)	*t*_20.285_ = 3.759	.**001** **
ASRM	1.5 (±2.19)	0.16 (±0.37)	*t*_20.170_ = 2.701	.**014** *
Information frequency^†^	3.60 (±1.19) MR: 21.90	3.16 (±1.02) MR: 18.00	*U* = 152.000	.296
COVID‐19 fears^‡^	3.98 (±2.39)	3.16 (±1.85)	*t*_37_ = 1.204	.236
Emotional distress^§^	1.13 (±1.05)	0.73 (±0.61)	*t*_37_ = 1.460	.153

Abbreviations: ASRM, Altman Self‐Rating Mania Scale; BD, bipolar disorder; BDI, Beck Depression Inventory; COPD, chronic obstructive lung disorder; HC, healthy controls; MR, mean rank; PSQI, Pittsburgh Sleeping Quality Index.

^†^ 1 = less than once a week, 2 = 1–3 times per week, 3 = 3–6 times per week, 4 = 1–3 times per day, 5 = 4–6 times per day, 6 = more than 6 times a day.

^‡^ “*How strongly do you rate your concerns and fears about the coronavirus?”, “How strongly do you rate your fear of contracting the coronavirus?” and “How strongly do you rate your fear of infecting others with the coronavirus?”* Mean of 3 0‐ to 10‐point scales.

^§^ “*Social distancing makes me feel lonely/ bored/ frustrated/ hopeless/ anxious”* Mean of 5 0‐ to 5‐point scales.

* *p* < .05

** *p* < .01.

Bonferroni correction for multiple comparisons for the PSQI components (0.05/7 tests): bold values indicate *p* < .0071 and are considered significant.

### 3.1 Sleep differences between BD and HC during strict and loosened restrictions

**FIGURE 1:**
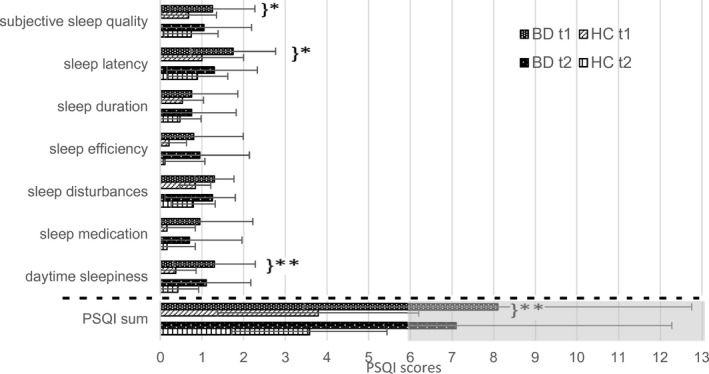
PSQI components at t1 and t2 in BD and HC. BD, bipolar disorder; HC, healthy controls; PSQI, Pittsburgh Sleeping Quality Index; gray underlaid = poor sleep in total; *p < .05; and **p < .01

A MANCOVA with age and sex as covariates showed significant group differences between the BD and the HC group in the sleep components and the sum of the PSQI at t1 (see Figure 1; *F*_7,29_ = 3.270, *p* = .011, η^2^ = 0.441). Patients with BD scored higher in all components which correspond to worse sleep. Significant differences were found in subjective sleep quality (*F*_1,35_ = 5.569, *p* = .024, η^2^ = 0.137), sleep latency (*F*_1,35_ = 4.305, *p* = .045, η^2^ = 0.110), daytime sleepiness (*F*_1,35_ = 8.195, *p* = .007, η^2^ = 0.190), the PSQI sum (*F*_1,35_ = 7.041, *p* = .012, η^2^ = 0.167), and a tendency in sleep disorders (*F*_1,35_ = 3.009, *p* = .092, η^2^ = 0.079).

At t2, the scores of the BD group were still higher, but there was no significant difference to HC anymore (*F*_9,27_ = 1.517, *p* = .192, η^2^ = 0.336). Individuals with BD scored 6 or above in PSQI sum at both times, which indicates a poor sleep. Standard deviations were higher at t1 than at t2.

### 3.2 Changes in sleep from April (strict ‐) to May (loosened restrictions)

The means in Figure 1 show an improvement in all sleep components from t1 to t2 in the BD group and almost all items in HC, but these changes were not statistically significant in paired *t* test analyses.

### 3.3 Correlations of sleep and information frequency as well as COVID‐19 fears

Within the BD group, in April (t1) the PSQI sum correlated significantly with information frequency (*r* = 0.603, *p = *.005) and COVID‐19 fears (*r* = 0.490, *p* = .039), but not with emotional distress (*r* = 0.301, *p = *.225). Apart from sleep duration significantly correlating with information frequency (*r* = 0.593, *p* = .006), no significant correlation between information frequency as well as COVID‐19 fears and the PSQI components was found after Bonferroni correction.

There were found large significant correlations in May (t2) between the PSQI sum score and information frequency (*r* = 0.541, *p = *.014), COVID‐19 fears (*r* = 0.536, *p = *.022), and emotional distress (*r* = 0.593, *p = *.009) in the BD group. There were multiple relations between PSQI components and COVID‐19‐related attitudes, but after Bonferroni correction, only the correlation between daytime sleepiness and COVID‐19 fears remained significant (*r* = 0.640, *p = *.004).

Within the HC group, we found only a few significant correlations. Analyses showed that lower information frequency was associated with more sleep disturbances at t1 (*r* = −0.597, *p* = .007). Moreover, the higher COVID‐19 fear scores were at t2, the worse the sleep efficiency was (*r* = 0.737, *p* = .001).

Furthermore, in the BD group, there were significant correlations of PSQI sum at t2 and information frequency at t1 (*r* = 0.712, *p = <*0.001) as well as COVID‐19 fears at t1 (*r* = 0.561, *p = *.015) and some sleep components after Bonferroni correction (subjective sleep quality and information frequency: *r* = 0.609, *p = *.004; subjective sleep quality and COVID‐19 fears: *r* = 0.644, *p = *.004; daytime sleepiness and COVID‐19 fears: *r* = 0.616, *p = *.006). Therefore, these variables were used for regression analyses.

### 3.4 Regression models of information frequency and COVID‐19 fears at t1 and sleep at t2

Tables 2 and 3 show the results of the linear regression models. Age and sex were also used as variables but did not explain any variance. Information frequency explained a significant proportion of variance in PSQI sum, subjective sleep quality, sleep duration, and sleep efficiency. COVID‐19 fears at t1 predicted the PSQI sum score as well as subjective sleep quality and daytime sleepiness at t2 with a high variance.

**TABLE 2:** Results from linear regression with information frequency about COVID‐19 from April 2020 as predictor of PSQI components in May 2020 in patients with BD adjusted for age and sex

	Model		Unstandardized coefficients		Standardized coefficients		*p* value
	*R* ^2^	*F*_3,19_	*B*	*SE*	*ß*	*t*	
PSQI sum	0.533	6.081	4.211	1.030	0.721	4.086	.**001** **
Subjective sleep quality	0.504	5.421	0.945	0.235	0.732	4.024	.**001** **
Sleep latency	0.217	1.477	0.505	0.266	0.434	1.901	.075
Sleep duration	0.601	8.019	0.797	0.197	0.661	4.050	.**001** **
Sleep efficiency	0.407	3.662	0.883	0.267	0.657	3.307	.**004** **
Sleep disturbances	0.293	2.214	0.190	0.135	0.306	1.412	.177
Sleep medication	0.171	1.104	0.180	0.334	0.127	0.540	.597
Daytime sleepiness	0.339	2.740	0.711	0.253	0.589	2.806	.013*

Note

Bonferroni correction for multiple comparisons for the PSQI components: threshold of significance: *p* < .0071 (0.05/7 tests) are marked in bold letters.

Abbreviations: BD, bipolar disorder; PSQI, Pittsburgh Sleeping Quality Index.

* *p* < .05

** *p* < .01.

**TABLE 3:** Results from linear regression with COVID‐19 fears from April 2020 as predictor of PSQI components in May 2020 in patients with BD adjusted for age and sex

	Model		Unstandardized coefficients		Standardized coefficients		*p* value
	*R* ^2^	*F*_3/19_	*B*	*SE*	*ß*	*t*	
PSQI sum	0.252	6.073	0.968	0.393	0.502	2.464	.**024** *
Subjective sleep quality	0.416	3.792	0.280	0.083	0.657	3.364	.**004** **
Sleep latency	0.157	0.996	0.134	0.090	0.350	1.493	.155
Sleep duration	0.401	3.564	0.186	0.079	0.468	2.365	.031*
Sleep efficiency	0.173	1.114	0.187	0.103	0.423	1.818	.088
Sleep disturbances	0.589	2.838	0.079	0.042	0.385	1.866	.081
Sleep medication	0.162	1.032	−0.037	0.110	−0.078	−0.334	.743
Daytime sleepiness	0.388	3.388	0.249	0.080	0.625	3.128	.**006** **

Note

Bonferroni correction for multiple comparisons for the PSQI components: threshold of significance: *p* < .0071 (0.05/7 tests) are marked in bold letters.

Abbreviations: BD, bipolar disorder; PSQI, Pittsburgh Sleeping Quality Index.

* *p* < .05

** *p* < .01.

## 4 DISCUSSION

The present study investigated sleep characteristics in individuals with BD in comparison with HC during the COVID‐19 pandemic in Austria. The results show overall poor sleep in the group with BD and worse sleep scores compared to HC. Individuals with BD informed themselves more frequently about the pandemic and had more emotional distress related to the social distancing in April when restrictions were very strict. In May, when legal regulations were loosened, the significant differences between the groups regarding information frequency, emotional distress, and sleep disappeared. At both points in time, BD individuals' sleep was worse, the more they informed themselves about the virus. Moreover COVID‐19‐related fears—including fears about the virus generally, contracting it themselves or infection of loved ones correlated with poor sleep. High information frequency and more COVID‐19‐related fears in April predicted sleep in May, in particular subjective sleep quality, sleep duration, sleep efficiency, and daytime sleepiness.

Individuals with BD reported worse sleeping habits than HC during the pandemic, in particular during very rigorous restrictions. This was independent of age and sex. Especially impairments in subjective sleep quality and sleep latency, daytime sleepiness, and sleep disorders were observed. This confirms findings from nonpandemic times. However, when regulations were less strict, the difference between the groups was not statistically significant anymore. As median PSQI scores showed, individuals with BD slept poorly at both points in time, whereas healthy controls reported good sleep in both April and May. This could be a consequence of depressive symptomatology found in BD, generally impaired sleep quality in BD even in euthymia or a warning symptom. As poor sleep leads to a decrease of functioning, concentration problems, stress, dissatisfaction, and an overall decreased quality of life, it is a serious problem for this vulnerable group (Slyepchenko et al., 2019). Among other biological mechanisms, the suprachiasmatic nucleus of the hypothalamus and the paraventricular nucleus of the thalamus control the sleep–wake rhythm. Studies have found altered morphologies in patients with BD (Manaye et al., 2005). Moreover, the clock genes play an essential role in BD as well as in the sleep–wake rhythm (Bengesser et al., 2018). The circadian clock affects a variety of physiological processes such as cardiovascular activity, sleep and alertness, metabolism, and brain function (Lyall et al., 2018).

The aim of the treatment of BD is the prevention of severe illness episodes as well as supporting a good psychosocial functioning and quality of life. The psychosocial consequences of BD could be decreased well‐being, stigma, cognitive dysfunction, loss of work, conflicts and disturbances in families, financial problems and suicide attempts. As sleep disturbances can trigger illness episodes and situations like the pandemic—as shown by this investigation—can negatively influence sleep, the treatment of these is one essential part of the multifactorial therapy concept. Direct interventions can impact sleep. For example, relaxation exercises in the evening support falling asleep. It is recommended to adhere to sleep hygiene like: regular sleeping times, regularity during the day, no sleep at daytimes, no heavy meals in the late evening, restricted alcohol consumption, and pleasant atmosphere in the sleeping room. Also, physical activity in daylight, in particular not in the late evening, positively influences sleep (Hartescu et al., 2015). Psychotherapeutic interventions such as cognitive behavioral therapy (CBT) and hypnotherapy have been shown to be beneficial for sleep disturbances (Friedrich et al., 2018). A special development for CBT‐insomnia (CBT‐I) was evaluated as more effective than psychoeducation only in BD (Harvey et al., 2015). In addition, the mindfulness‐based approach to the treatment of insomnia (MBT‐I) is based on interventions aiming to increase emotion regulation and to reduce stress and proved to be efficient to treat sleeping disorders as well (Ong & Sholtes, 2010). If this is not sufficient, the use of short‐term sleeping medication or medium‐term sedating antidepressive drugs is recommended to treat sleep disturbances. Moreover, the intestinal‐brain axis is related to sleep via various mechanisms such as intestinal permeability, immune system activation, inflammation, energy harvest, and bacterial diversity (Wagner‐Skacel et al., 2020). Consequently, diet impacts sleep as well (St‐Onge et al., 2016).

Even if not statistically significant, an improvement from April to May of all sleep components in both groups was obvious regarding the means. The values of the different PSQI parameters varied a little bit more at t1, particularly in the BD group. The reason for this might be that some individuals with BD had more difficulties adjusting to this extraordinary situation in April. Then, an adaption of the situation and a reduction of the initially complete uncertainty might have improved sleep problems of this particular group and approximate to the individuals, who slept better the whole time. Moreover, since the loosened restrictions people were allowed to live a less restricted and little more normalized life. Furthermore, it is conceivable that fewer working hours, fewer appointments, and more time for physical activity in daylight might have had a positive effect on sleep (Altena et al., 2020). Then again, having more time might correlate with gathering more information resulting in rumination and worse sleep. Contrary Some people were able to adhere to a sleep–wake rhythm which corresponds more to their endogenous morning or evening type while working from home, which supports good sleep (Wheaton et al., 2016). Moreover, our results show that COVID‐19‐related fears and information frequency also have a considerable impact on sleep a month later.

The information frequency in the beginning of the pandemic was quite high, as the mean of informing oneself was higher than once a day. In the second month, the frequency decreased. A study in China showed that people who spent more time focusing on information about COVID‐19 had a higher prevalence for generalized anxiety disorder (Huang & Zhao, 2020). Individuals with BD informed themselves even more than HC, which could be explained by a different lifestyle. In particular, in April the uncertainty about the virus, the disease and the association with somatic and mental comorbidities was immense. Physical contact with others in order to validate one's feelings and opinion was not possible. Furthermore, it was unclear whether patients with mental disorders could get their usual psychosocial treatment including regular appointments in the outpatient setting due to social distancing regulations. Presumably, the biggest burden was not knowing how the future will unfold. Obtaining informing about infection rates, case of deaths and legal regulations might have increased individuals' feeling of certainty and control. However, our results also show that higher information frequency was related to worse sleep in the bipolar cohort during strict and loosened restrictions. As the information frequency decreased over time and with it subjective sleep quality, sleep duration, and sleep efficiency improved, the hope remains that sleep disturbances will be getting better in follow‐up investigations in this vulnerable group of BD. Potentially, the mentally healthy people in our cohort could cope more effectively with the flood of information. In our cohort significant more HC had to work in April and therefore had a structured day. Consequently, individuals with BD had more time for informing themselves and think about pandemic‐related topics. Possibly, the permanent engagement with COVID‐19‐related topics resulted in rumination and, subsequently, poor sleep, especially for the BD group. The WHO officially recommends to minimize watching, reading or listening to news about COVID‐19 to once or twice a day. Everybody should aim to be informed just enough to be able to act responsibly (World Health Organization, 2020). The same was advised for individuals with BD (Siqueira et al., 2020).

Individuals with BD as well as HC had concerns and fears about the virus, their own health and infection of loved ones to a moderate extent. The emotional distress due to social distancing was rather low in our study in both groups. The findings show associations of COVID‐19‐related fears with poor sleep in April and May. Moreover, fears in the early stages predict subjective sleep quality and daytime sleepiness in the follow‐up. We therefore recommend to include acknowledging COVID‐19‐related fears in psychotherapy of BD, addressing not only psychosocial symptoms such as mood, but also sleep. However, our results indicate that fears of the virus in general have a bigger impact on sleep than social distancing measured with the emotional distress variable. The task force of the European CBT Academy recommends applying common sleep hygiene during the pandemic. Moreover, they advise using the opportunity of following the endogenous sleeping type and adapted CBT methods if possible in home office (Altena et al., 2020). Stefana et al. (2020) stated that the pandemic could also be an opportunity for new therapeutic ways, such as mindfulness trainings, sleep hygiene, psychoeducation or even pharmaceutical prescription, and psychotherapy can be offered online or via telephone. Social media interaction grew in times of restrictions, but presumably this is not equally valuable as face‐to‐face contact (Altena et al., 2020). In sum, our results make a considerable contribution when developing future treatment approaches for BD during pandemics or crises. Further studies should investigate the impact of online communication on sleeping disorders associated with loneliness and depression.

### 4.1 Limitations

There are several limitations of this study. Due to the online design, no objective rating of current psychopathological symptoms was assessed. Furthermore, COVID‐19‐related fears and emotional distress were self‐conducted variables with no reference value. Additionally, the sample size is rather small, but therefore we had complete data of almost as many patients as HC over two points of measurement. Due to the clinical design, the groups differed in age, which was adjusted in the statistical analyses. Finally, we do not have pre‐COVID‐19 baseline measures of mood and sleep quality in our participants.

## 5 CONCLUSIONS

Austrian individuals with BD reported sleeping more poorly than HC during the COVID‐19 pandemic in April 2020. Individuals' sleep in May 2020—in particular worse subjective sleep quality, sleep duration, sleep efficiency, and daytime sleepiness—could be predicted by higher information frequency, increased fears of the virus generally, and either oneself or others getting infected. Good sleep is essential for functioning and well‐being, especially for the vulnerable BD group. Therefore, it is important that information about the pandemic is gathered to a reasonable extent and mental health professionals include COVID‐19‐related fears when currently treating bipolar individuals.

### PEER REVIEW

The peer review history for this article is available at https://publons.com/publon/10.1002/brb3.2182.
